# Supplementary material for: Circulation of Dengue Virus Serotype 1 Genotype V and Dengue Virus Serotype 2 Genotype III in Tocantins State, Northern Brazil, 2021–2022
Source: Viruses. 2023 Oct 24;15(11):2136. doi: 10.3390/v15112136 (PMC10674946; doi:10.3390/v15112136)
Supplement: Supplementary file 1 [file viruses-15-02136-s001.zip › viruses-2641652-supplementary/viruses-2641652-supplementary/Figures S1 to S4.pdf]

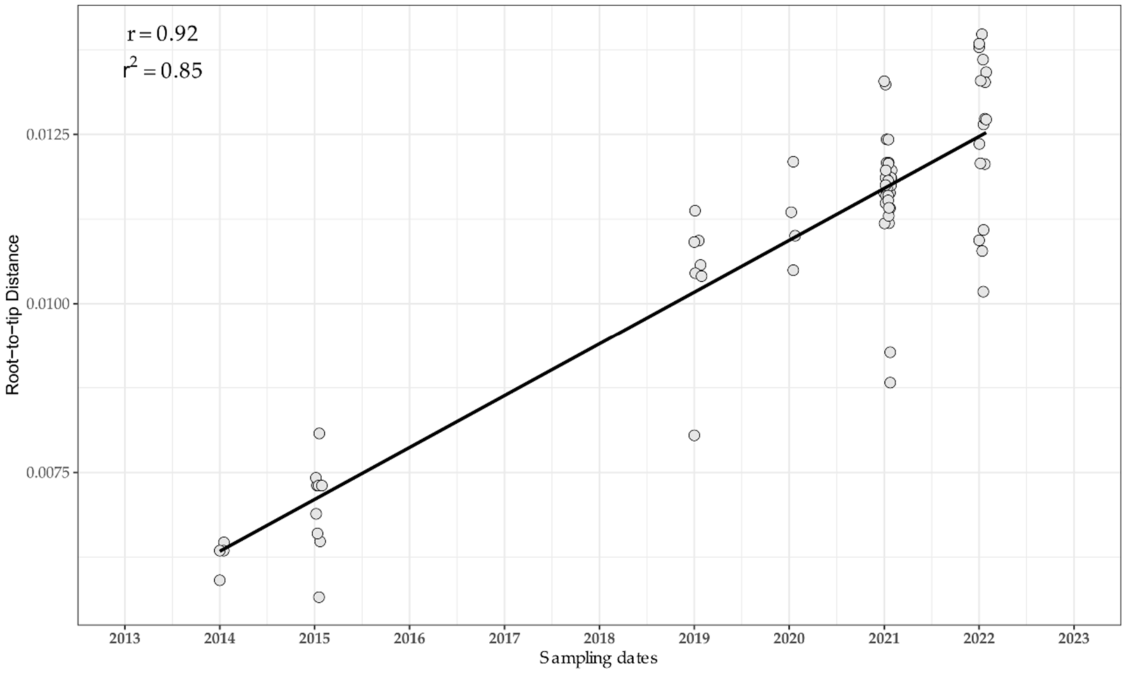

Figure S1. Root-to-tip regression of genetic distances and sampling dates for 76 complete and near-complete Clade I of DENV-1 Genotype V sequences sampled in Brazil, South America and North America. Correlation coefficient (r) and r squared are depicted above the graph.

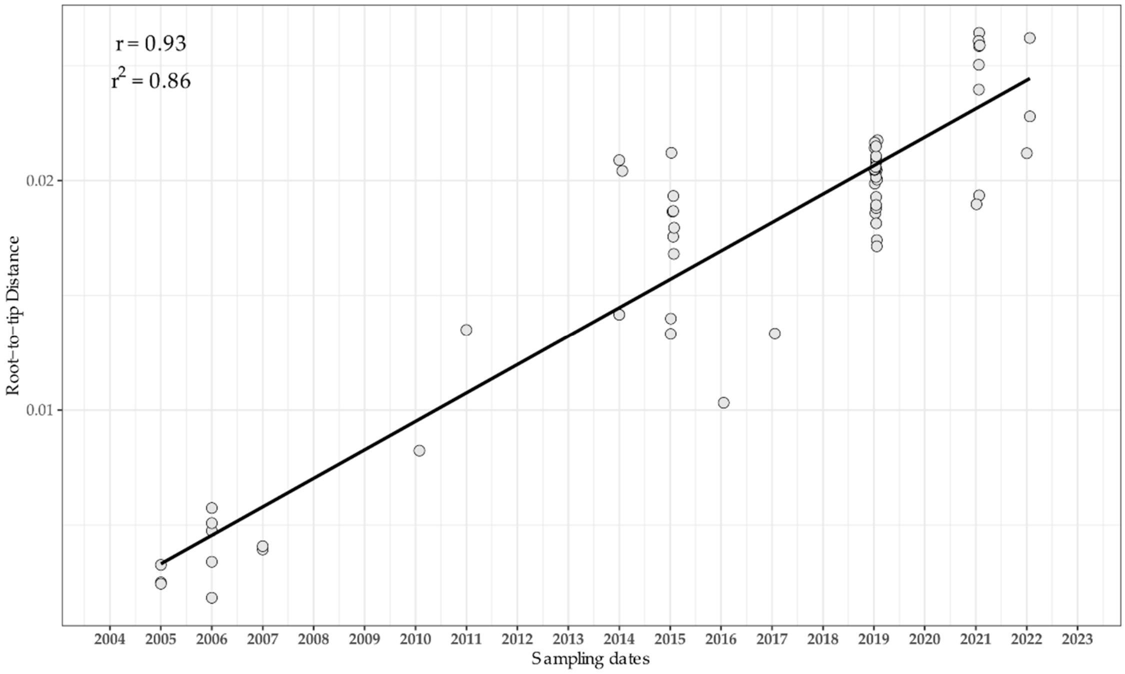

Figure S2. Root-to-tip regression of genetic distances and sampling dates for 63 complete and near-complete Clade II of DENV-1 Genotype V sequences sampled in Brazil and South America. Correlation coefficient (r) and r squared are depicted above the graph.

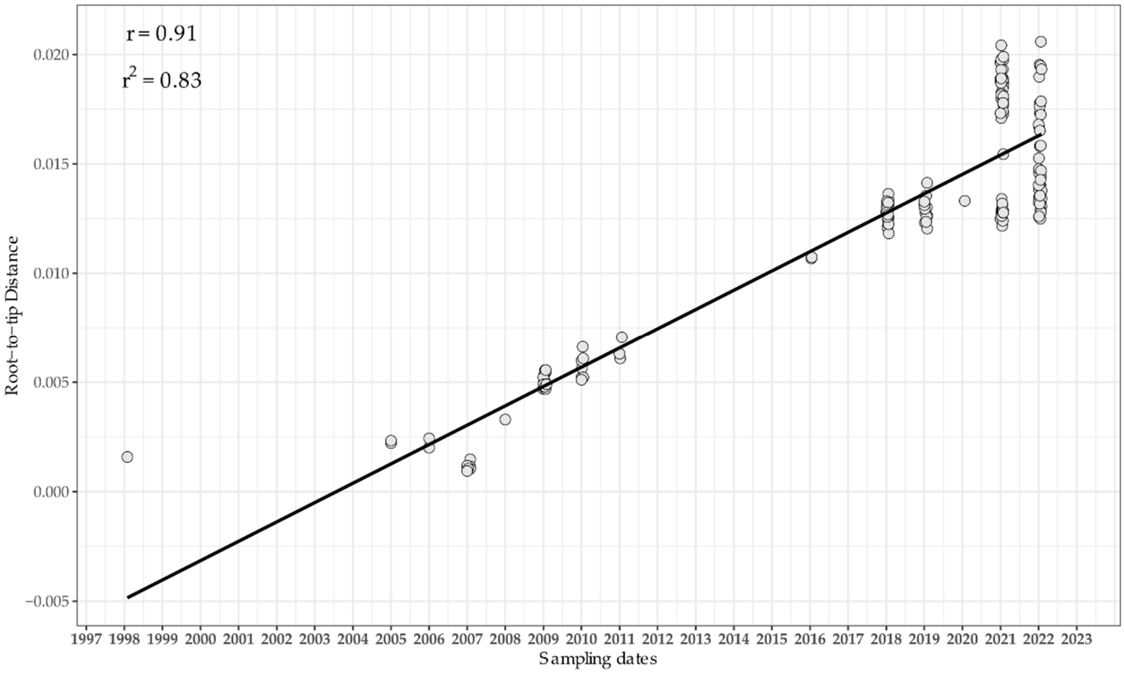

Figure S3. Root-to-tip regression of genetic distances and sampling dates for 170 complete and near-complete Clade III of DENV-1 Genotype V sequences sampled in Brazil, South America and North America. Correlation coefficient (r) and r squared are depicted above the graph.

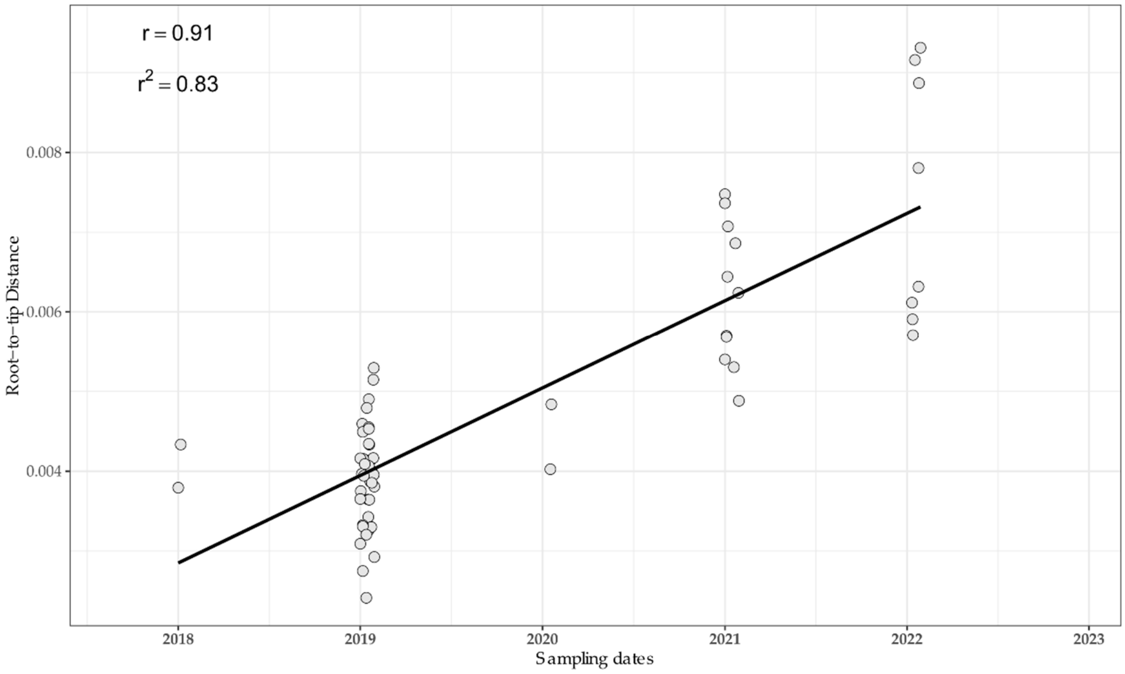

Figure S4. Root-to-tip regression of genetic distances and sampling dates for 63 complete and near-complete of DENV-2 sequences sampled in Brazil. Correlation coefficient (r) and r squared are depicted above the graph.
